# Obstetric Emergency Supply Chain Dynamics and Information Flow Among Obstetric Emergency Supply Chain Employees: Key Informant Interview Study

**DOI:** 10.2196/59690

**Published:** 2024-09-05

**Authors:** Kylie Dougherty, Abebe Gebremariam, Heran Biza, Mulusew Belew, Natalie Benda, Yihenew Tesfaye, John Cranmer, Suzanne Bakken

**Affiliations:** 1 Department of Medical Social Sciences Northwestern University Feinberg School of Medicine Chicago, IL United States; 2 Woodruff Health Sciences Center Emory University Atlanta, GA United States; 3 Emory-Ethiopia Partnership Bahir Dar Ethiopia; 4 Nell Hodgson Woodruff School of Nursing Emory University Atlanta, GA United States; 5 College of Health Sciences Bahir Dar University Bahir Dar Ethiopia; 6 School of Nursing Columbia University New York, NY United States; 7 Center for the Study of Human Health Emory University Atlanta, GA United States

**Keywords:** basic emergency obstetric care needs, BEmOC, supply chain, Ethiopia, Sociotechnical Model

## Abstract

**Background:**

For the past several decades, the Ethiopian Ministry of Health has worked to decrease the maternal mortality ratio (MMR)—the number of pregnant women dying per 100,000 live births. However, with the most recently reported MMR of 267, Ethiopia still ranks high in the MMR globally and needs additional interventions to lower the MMR to achieve the sustainable development goal of 70. One factor contributing to the current MMR is the frequent stockouts of critical medications and supplies needed to treat obstetric emergencies.

**Objective:**

This study describes the obstetric emergency supply chain (OESC) dynamics and information flow in Amhara, Ethiopia, as a crucial first step in closing stockouts and gaps in supply availability.

**Methods:**

Applying qualitative descriptive methodology, the research team performed 17 semistructured interviews with employees of the OESC at the federal, regional, and facility level to describe and gain an understanding of the system in the region, communication flow, and current barriers and facilitators to consistent emergency supply availability. The team performed inductive and deductive analysis and used the “Sociotechnical Model for Studying Health Information Technology in Complex Adaptive Healthcare Systems” to guide the deductive portion.

**Results:**

The interviews identified several locations within the OESC where barriers could be addressed to improve overall facility-level readiness, such as gaps in communication about supply needs and availability in health care facilities and regional supply hubs and a lack of data transparency at the facility level. Ordering supplies through the integrated pharmaceutical logistics system was a well-established process and a frequently noted strength. Furthermore, having inventory data in one place was a benefit to pharmacists and supply managers who would need to use the data to determine their historic consumption. The greatest concern related to the workflow and communication of the OESC was an inability to accurately forecast future supply needs. This is a critical issue because inaccurate forecasting can lead to undersupplying and stockouts or oversupplying and waste of medication due to expiration.

**Conclusions:**

As a result of these interviews, we gained a nuanced understanding of the information needs for various levels of the health system to maintain a consistent supply of obstetric emergency resources and ultimately increase maternal survival. This study’s findings will inform future work to create customized strategies that increase supply availability in facilities and the region overall, specifically the development of electronic dashboards to increase data availability at the regional and facility levels. Without comprehensive and timely data about the OESC, facilities will continue to remain in the dark about their true readiness to manage basic obstetric emergencies, and the central Ethiopian Pharmaceutical Supply Service and regional hubs will not have the necessary information to provide essential emergency supplies prospectively before stockouts and maternal deaths occur.

## Introduction

### Background

For the past several decades, the Ethiopian Ministry of Health (MOH) has worked to decrease the maternal mortality ratio (MMR)—the number of pregnant mothers dying per 100,000 live births [1]. However, with the most recently reported MMR of 267, Ethiopia still ranks high in the MMR globally and needs additional interventions to lower the MMR to the sustainable development goal of 70 [2]. Therefore, the Ethiopian MOH is focusing on improving “the health systems capacity to offer quality care that meets women’s needs (the supply side)” [3,4]. Toward this goal, the Amhara Regional Health Bureau in Amhara and the Ethiopian MOH have identified the need for a real-time obstetric emergency readiness system, which will assist in measuring and monitoring facility-level readiness to manage the 6 most common basic emergency obstetric care needs (BEmOC), including a set of core drugs; commodities; and resources to identify emergencies, treat them, and monitor-modify therapy as clinically indicated [5].

Formative work to articulate the specific needs, barriers, and facilitators for the development and implementation of a real-time obstetric emergency readiness system is foundational to meeting required needs and ensuring that the system matches the environment in which it is deployed [6].

### Objective

This study aimed to describe the obstetric emergency supply chain (OESC) dynamics and information flow through semistructured qualitative interviews with key informants from different levels of Ethiopia’s health care system. This information would be used to guide the design and eventual implementation of electronic dashboards as a component of the real-time obstetric emergency readiness system.

## Methods

### Theoretical Model

**Figure 1:**
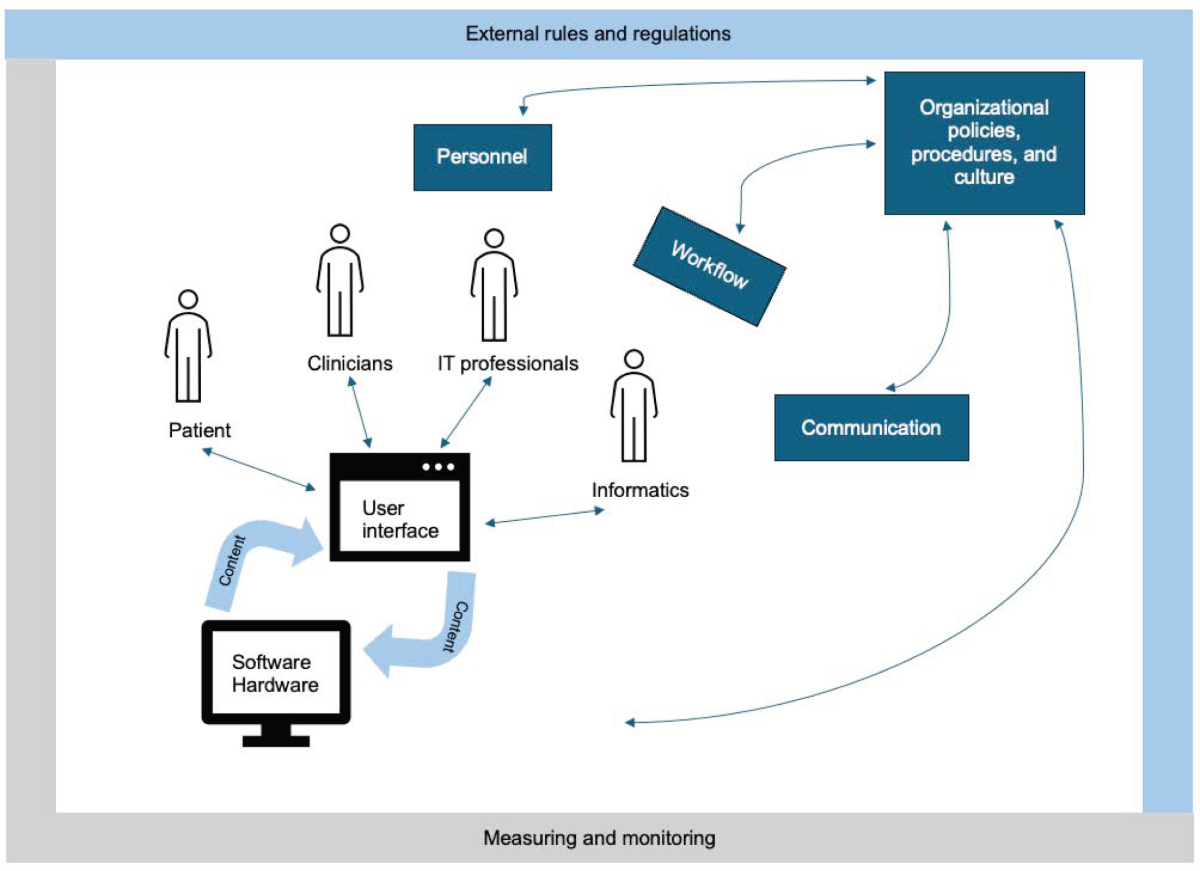
Sociotechnical Model for Studying Health Information Technology in Complex Adaptive Healthcare Systems.

The “Sociotechnical Model for Studying Health Information Technology in Complex Adaptive Healthcare Systems” guided this study (Figure 1) [7]. The Sociotechnical Model is a dynamic and interconnected model that provides a thorough picture of the process for designing, implementing, and evaluating health information technology (HIT) through 8 interconnected dimensions: *hardware and software, clinical content, human-computer interface, people, workflow and communication, internal organization features, external rules and regulations,* and *measuring and monitoring* (Textbox 1) [7]*.*

**Sociotechnical Model for Studying Health Information Technology in Complex Adaptive Healthcare Systems dimensions and definitions.**

**Model dimension and definitions**

- Hardware and software
  The digital infrastructure and equipment that is used to operationalize the clinical application being explored
- Clinical content
  The categorical or numerical data and images that make up the “language” of the clinical application
- Human-computer interface
  All aspects of the digital application that the user can see, touch, hear, or manipulate
- People
  The application users from the developers of the health information technology (HIT) to the end users
- Workflow and communication
  The necessary steps that a user must accomplish to successfully and effectively complete the task at hand
- Internal organizational features
  The policies, procedures, and culture within the specific organization using the HIT
- External rules and regulations
  The policies, procedures, and culture within the larger geographic location where the HIT is located
- Measuring and monitoring
  The evaluation process and method of measuring the effectiveness of the HIT change, including both intended and unintended consequences

### Guiding Methodology

Guided by qualitative descriptive methodology, we conducted semistructured interviews to further describe and contextualize the gaps in emergency supply availability discovered in an earlier quantitative analysis of facility-level obstetric emergency readiness in the region [8,9]. The interviews explored multifactorial causes of emergency commodity stockouts, the current health system approaches to restocking commodities (from the federal, regional, and facility level), and information flow gaps and dynamics that could contribute to emergency supply stockouts. Data collection and analysis occurred concurrently [10].

### Recruitment and Sample

We recruited a purposive sample of OESC experts from across the federal, regional, and facility level of care in Ethiopia. This included federal MOH government officials working in supply chain management and maternal survival in Addis Ababa, regional health bureau officials with obstetric expertise, regional pharmaceutical supply system employees, and supply managers and pharmacists from individual health care facilities. To participate in this study, individuals had to be at least 18 years of age, speak English and Amharic, and be full-time employees in one aspect of Amhara’s OESC.

### Ethical Considerations

The study was approved by institutional review boards at Columbia University (IRB-AAAU2006) and Emory University (MOD005-STUDY00005335) and received ethics approval from Amhara Public Health Institute (NoH/RfftT1D1o7144). All participants provided verbal consent before participation. All data were deidentified before data analysis. Participants received no compensation for their participation in this study.

### Data Collection: Semistructured Interviews

We created an interview guide with open-ended questions to explore the flow of information through the OESC, including barriers and facilitators (factors that enhance or impede access and use) with paper-based systems and the computer-based integrated pharmaceutical logistics system (IPLS; Textbox 2 and Multimedia Appendix 1). We conducted interviews in English (federal level) and Amharic (regional and facility level). The interviews were audio recorded and lasted approximately 60 minutes. Amharic is the national language, and individuals working in Ethiopia’s health care field received their training and education in English. During the interviews, we performed member checks by summarizing the main concepts and ideas the participants voiced and asked for confirmation to ensure we accurately captured their perspectives [11].

**Sample interview guide questions for semistructured qualitative interviews with obstetric emergency supply chain employees in Amhara, Ethiopia.**

**Questions**

- What do you see as the major challenges to having the right supplies on hand to deal with obstetric emergencies when they occur?
  (Communication breakdowns, frequently unable to obtain certain items, computers available, or consistent Wi-Fi)
- Can you describe your process for ordering and shipping obstetric emergency supplies?
  (Barriers and facilitators in the process, how often it occurs, who does this task, was there any training, and decision-making process)
- What are your impressions of the paper-based supply request system?
  (Likes, dislikes, barriers, and facilitators for use, and areas of improvement)
- Can you describe your experience using the integrated pharmaceutical logistics system?
  (Likes, dislikes, barriers, and facilitators for use, areas of improvement, and training if it occurred)
- Is there anything else that you’d like me to know about the topics that we’ve discussed today that I didn’t ask about?

### Data Management and Preparation

We stored audio files in OneDrive (Microsoft Corporation), an encrypted, password-protected electronic site accessible only to the research team members [12]. All interviewees were identified by a participant number linked to the OESC level in which that individual worked (ie, federal, regional, or facility level). The participant numbers were linked to their names and stored behind an encrypted, password-protected site. We checked the transcripts for accuracy against the audio recording in English or Amharic and deidentified them before sharing them with other research team members. We used ATLAS.ti (ATLAS.ti GmbH version 9) to manage, code, and analyze all qualitative data [13].

### Data Analysis

We performed directed content (ie, deductive) and inductive analysis of the interview transcripts. The directed content analysis was guided by a codebook developed by KD and SB (Multimedia Appendix 2) based on the a priori constructs of the Sociotechnical Model dimensions [7,14]. During data analysis, we added codes to the *human-computer interface* dimension of the codebook to reflect the emergence of additional themes. KD independently coded the transcripts, generated themes, and documented the coding. SB reviewed the documentation and refined codes with KD to establish the confirmability of the coding decisions. We created saturation tables using a priori constructs of the Sociotechnical Model, concurrently with data analysis to document if and when data saturation, a measure of data adequacy that signals that no further data collection is needed, had occurred (Multimedia Appendix 3) [15]. We specifically used a priori thematic saturation to guide the decision on when to stop data collection [16].

## Results

### Overview

We conducted 17 interviews (federal level: n=5, 29%; regional level: n=5, 29%; facility level: n=7, 41%) from February 17 to March 17, 2023. We relied on the qualitative research experience of the data collectors (KD and YA) and data saturation (Multimedia Appendix 3) to guide the decision on when to stop participant recruitment. The sample was predominantly male (14/17, 82%), with an age range of 30 to 60 years, and with 2 to 38 years of experience working within the OESC (Multimedia Appendix 4). While we analyzed all 8 dimensions of the Sociotechnical Model, in the main paper, we focused on the 5 (62%) dimensions most pertinent to the development of dashboards for monitoring facility-level readiness to manage obstetric emergencies and reported on the other 3 (38%) in Multimedia Appendix 5.

### Hardware and Software

In 2014, Ethiopia launched IPLS which allows facilities to place orders for medical supplies either electronically or through paper forms. IPLS also enables the central Ethiopian Pharmaceutical Supply Service (EPSS) and regional hubs to see reports on supply availability at the regional and national levels.

Two software systems are used within the OESC to monitor the movement of medical supplies, namely *Vitas* and *Dagu*. *Vitas* is used by central EPSS, which supplies the country with medications and medical supplies. The director general of EPSS and input officers have access to *Vitas* dashboards, which allows them to check supply availability and track commodities at both the federal and regional hub levels. *Dagu* is the software used at individual health care facilities. *Dagu* is used at the facility level to monitor human resources and service availability, but it does not have dashboards for medical supply logistics and movement. Some software in the OESC have components that function offline while other features are fully dependent on live internet access. Barriers (eg, unstable internet access and frequent electric power outages) and facilitators (eg, some IPLS components available offline) and related quotes are summarized in Table 1.

**Table 1:** Reported barriers and facilitators for the hardware and software dimension (the digital infrastructure and equipment that is used to operational the clinical application being explored) of the Sociotechnical Model for Studying Health Information Technology in Complex Adaptive Healthcare Systems extracted from semistructured interviews with obstetric emergency supply chain employees in Amhara, Ethiopia.

Themes		Quotes
**Barriers**		
	Lack of or outdated laptops, tablets, and phones	“Most health facilities lack a computer, a printer, or the training required to actively utilize the [current electronic system(s)].” [Regional #1] “Updating our computers and laptops would be a good idea since we’ve been using them for a decade! It’s tough to get them to work in this setting.” [Regional #4]
	Unstable and inaccessible internet	“In general, 75% of the facilities have no internet access.” [Federal #2]
	Technology is not tailored for use on mobile phones or offline	“There are many things that need to be updated. Many systems have changed, but they are not keeping up with the change. For example, we have to find information on our mobile phones. But there is not much like that. It should be transparent.” [Facility #3] “I want the IPLS system to be mobile application-based; if it is, then EPSS or other health offices should be checking the... hospital system.” [Facility #7]
**Facilitators**		
	Tablets provided to some facilities	“We distributed tablets to all health posts to track the workflow of health posts as well as any commodity-related issue activities.” [Federal #2]
	Most employees have access to mobile phones	“Almost all health extension workers have a mobile phone and can easily see any piece of information.” [Federal #2]
	Some IPLS^a^ components can work offline if a source of electrical power is available	“In some areas, the facilities are using solar panels.” [Federal #2]

^a^IPLS: integrated pharmaceutical logistics system.

### Clinical Content

Respondents at all levels of the supply chain agreed that several pieces of information are critical to ensure facilities are ready to provide BEmOC and to prevent emergency supply stockouts. In many health care facilities, pharmacists hand-calculate the last month’s consumption, average monthly consumption, and they forecast future monthly and annual supply demands. In contrast, some health care facilities used components of the electronic IPLS to calculate expiration and current quantity of supplies automatically. These respondents stated that the automatic calculations assisted in their consumption calculations. Overall, participants reported a desire for an automated system at all health care facilities that will calculate consumption, waste, and medication expiration in real time.

Upon receiving orders from hubs, health care facilities must know and report the number of items they originally ordered, the number of items received, the number of items missing from the order, who sent and received the shipment, and the date the supplies arrived at the health care facility. These pieces of information are stored in various data sources, such as bin cards and stock record cards resulting in a fragmented, nontrackable, and nonintegrated reporting system.

Furthermore, when individual health care facilities are determining how much medicine and medical supplies to order, they must also know the disease burden in the population that they serve, and which items are available for ordering. For example, in obstetric emergencies, facilities should know the estimated size of their catchment population, the number of mothers who give birth at their facility for antenatal care service, the number of pregnant women who attend the facility, and the number of mothers who are typically referred to higher facility levels for more intensive treatment. These complex variables are implicitly factored into health care facility orders, but there is no system to formally account for those variables in creating orders from facilities. Respondent perceptions of barriers (eg, lack of reliable, high-quality, real-time consumption data) and facilitators (eg, integration of medical supplies into one place) as well as exemplar quotes are displayed in Table 2.

**Table 2:** Reported barriers and facilitators for the clinical content dimension (the categorical or numerical data and images that make up the language of the clinical application) of the Sociotechnical Model for Studying Health Information Technology in Complex Adaptive Healthcare Systems extracted from semistructured interviews with obstetric emergency supply chain employees in Amhara, Ethiopia.

Themes			Quotes
**Barriers**			
	Lack of accurate, high-quality consumption data	“When we go to [look at] the health facility level, especially the primary centers like health posts and health centers, it is very difficult to visualize what they have or do not have.” [Federal #2] “The expert tries to send information by speculation; this is also another problem because you may take more medication and supplies than you need, which will lead to expired medication and supplies and sometimes the institution also faces a shortage of medication and supplies because they send false information. Thus, using guessing information will create overstock and understock problems.” [Regional #1] “Accurate and on time information is highly needed and very vital to get the right supplies on hand to deal with problems, but unfortunately, we don’t gather reliable information and we are making mistakes.” [Regional #1] “We are using the issue data as a proxy data [for] consumption and we usually use this data for the national quantification so it might exaggerate or decrease the national quantification.” [Federal #3] “The IPLS does not take the average monthly consumption component into account, necessitating a review of these issues.” [Facility #7] “There are also issues with the quality of data because there is no way for us to make sure the person sent a valid report with real information or not.” [Regional #2]	
**Facilitators**			
	High integration of medical supplies into 1 place	“Compared to the previous IPLS, the current IPLS manual system is highly integrated. The previous system was very scattered because the pharmacy sent requests by themselves, the laboratory sent requests by themselves, and others did the same thing, and it was very uncoordinated. As of right now, the integrated system is very good, and all requests are sent in the same direction and in a coordinated manner.” [Facility #2] “Most of our facilities are integrated into IPLS, particularly most maternal commodities, which were integrated into IPLS after the 2009 Ethiopian calendar (2017 Gregorian Calendar). Therefore, everyone can check what they have, what they do not have, what is nearing its expiration date.” [Federal #2] “Following EPSS’s establishment, the fragmented administration system completely stopped. For example, when we deliver medicines such as TB, HIV medicines, HIV examination kits, and mother-and-child-related medicines, we do so in an integrated way, not in fragments. When we deliver, we take all the HIV, TB, and medicines through one route because it is an IPLS system, which means we deliver integrated.” [Regional #4]	

### Workflow and Communication

In the Amhara region, there are 918 health centers, 103 hospitals, and 19 supply hubs. To determine the type and quantity of medical supplies to order, a facility must first determine its monthly consumption and average monthly consumption to forecast future needs. When requesting for supplies, facilities can request up to a maximum of 4 months of stock. The head of each department or unit at the health care facility plans monthly consumption every 2 weeks and submits an internal request and reporting forms to the central store or pharmacy in the facility. The pharmacist reviews and fulfills these biweekly requests from the facility’s stock and also completes and submits requesting and reporting forms (RRFs) to their designated regional supply hub to refill facility-level supplies. Pharmacists submit these forms every 1 to 2 months depending on the size of the population they serve and may also submit an emergency RRF if they have less than 2 weeks’ supply of an item or experience a stockout. The RRFs are either submitted in paper form or electronically through the IPLS. A health care facility–level respondent describes the critical role of IPLS saying, “It acts as the system’s brain and is crucial for having the right medication in the right quantity and of the right quality at the right time.”

After receiving the RRFs through the IPLS, regional hubs review the forms and if they have the stock on hand in the hub, they create a stock transfer voucher and fulfill the request by providing the commodity to the facility. In addition, the hub consolidates the consumption reports and RRFs for the individual health care facilities under their jurisdiction and estimates overall consumption and forecasting for the upcoming months, which they report in an RRF and send to the central or national office of the EPSS for fulfillment every 1 to 2 months.

**Figure 2:**
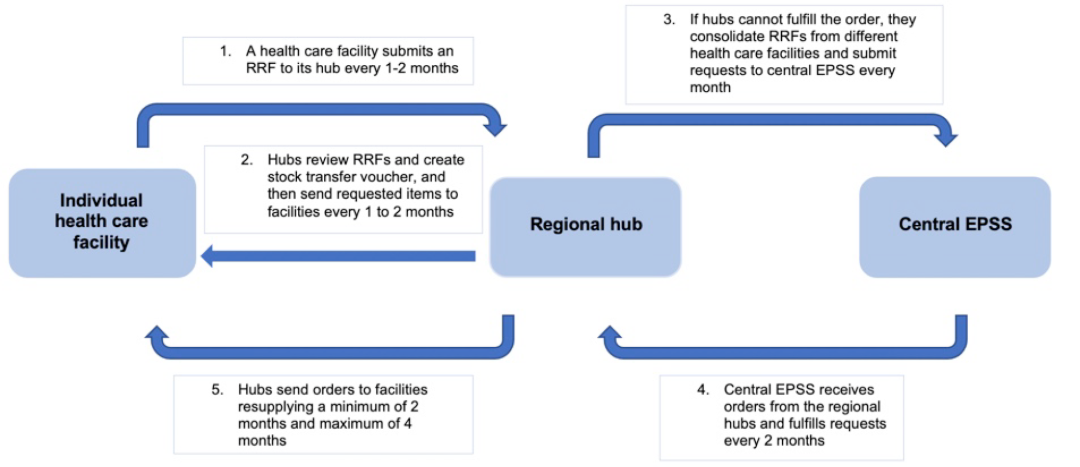
Procurement process for ordering and receiving medical supplies in Amhara, Ethiopia, from the facility level through regional hubs and at the central hub level. EPSS: Ethiopian Pharmaceutical Supply Service; RRF: requesting and reporting form.

Central EPSS reviews the RRFs from their hubs and fulfills the orders based on consumption reports and local and national forecasting numbers. The hubs receive the products from central EPSS and review the orders to see if they received the full or partial order. The hubs compare this information to the requests they have received from their facilities and send the supplies to the facilities every 1 to 2 months. After receiving the order from their designated hub, health care facilities then review what they have received and dispense it throughout the various departments or units at the facility. They track the new deliveries and existing inventory with bin cards and stock record cards that are paper-based. A summary of the various data sources and communication systems present in this supply chain is presented in Multimedia Appendix 6. Figure 2 illustrates the procurement process. The barriers and facilitators related to workflow and communication are presented in Table 3 with sample quotes from participants at various levels of the system.

**Table 3:** Reported barriers and facilitators for the workflow and communication dimension (the necessary steps that a user must accomplish to successfully and effectively complete the task at hand) of the Sociotechnical Model for Studying Health Information Technology in Complex Adaptive Healthcare Systems extracted from semistructured interviews with obstetric emergency supply chain employees in Amhara, Ethiopia.

Themes		Quotes
**Barriers**		
	Inaccurate forecasting	“The forecasting error at Ethiopia’s health facility is not better than 35%, and you see 40%, very good facilities are forecasting at 50%. What does this mean, they are either over-quantifying or under-quantifying.” [Federal #1] “The knowledge gap about capacity and forecasting error is evident.” [Federal #1] “There is a [communication] barrier, the hub knows only the stock on hand issued data, so as a logisticians, the issue data is the proxy data [for] consumption, that is not the actual consumption but we use it as a proxy data as consumption and we usually use this data for the national quantification so it might exaggerate or decrease the national quantification and the forecasting that might occur.” [Federal #3] “If we request medical equipment, we have to consider consumption, but currently, we request the amount of medical equipment by guessing, not based on consumption data, so this exposes us to medical equipment scarcity.” [Facility #2]
	Outdated forms (both paper and electronic)	“There is something that needs to be improved. I think it [IPLS] has not changed since the first format design. Therefore, the format must be adjusted, now the information network has grown.” [Facility #3]
	Overreliance on paper-based methods	“The other challenge is the system problem. Most of our health facilities use a manual method of requesting and ordering medical supplies and equipment.” [Regional #1] “If it is paper based, it is like to be blinded. It’s going to be difficult to find out the medicine that we are running out of stock, so it must be digital to analyze the data. Paper-based has nothing to show the expert how much demand there is today and what demand there will be tomorrow. Paper-based is not good. I think it should be updated.” [Facility #3] “Paper-based systems are not appropriate for the 21st century; they are archaic, tedious, difficult to access, and unsuitable for auditing.” [Facility #7] “In the current situation, the system between health facilities and us [regional hub] is still manual, so it’s also possible for us to make several mistakes when it comes to requesting medicines due to this manual system.” [Regional #4] “The paper-based or hard-copy system of supplying or requesting the information is very helpful, but it is very difficult to share information immediately.” [Regional #5]
**Facilitators**		
	There is an established process for ordering medical supplies through IPLS^a^	“The integrated system is very good, and all requests are sent in the same direction and in a coordinated manner.” [Facility #2]

^a^IPLS: integrated pharmaceutical logistics system.

### Human-Computer Interface

Individuals interact with the OESC at the federal, regional, and individual facility levels of the system through a variety of human-computer interfaces. Multimedia Appendix 7 describes the needs of the 3 types of users as well as how they interact with the interfaces available at their level of the health system. In addition to the current interfaces, some respondents wanted the electronic IPLS to be accessible via a mobile app or on mobile devices so that they have greater access to the data, particularly when desktop or laptop computers were not available at the health facilities and alternative mechanisms were needed to use the electronic components of IPLS. Multimedia Appendix 8 summarizes the barriers and facilitators identified for human-computer interfaces.

## Discussion

### Principal Findings

We used the Sociotechnical Model to explicate participants’ perceptions of barriers and facilitators to securing and managing supplies within the OESC in Amhara, Ethiopia, and to identify intervention targets to increase BEmOC supply availability at the facility level as a key step for reducing Ethiopia’s MMR [7]. Changing one component within the OESC can influence the success of the system, but current barriers may impede or interact with the system in unique and unintended ways. An example of this would be if the EPSS increased the number of computers and tablets available to health care facilities but failed to explore users’ usability and ease of use concerns. If the technology is still viewed as difficult to use and users continue to prefer paper-based forms, then EPSS as a whole will not see the full benefits of increasing access to the technology. This type of outcome is highlighted in a recent scoping review in low- and middle-income countries, which included Ethiopia, and it identified lack of training and user involvement as barriers to successful digital health interventions [17]. To ensure the success of the electronic components of IPLS the researchers and individuals working within this field can build on the preexisting strengths found in the IPLS to rectify barriers and concerns for HIT-based real-time monitoring of BEmOC supply provision and availability.

The lack of access to computers and tablets led to lower use of the electronic components of IPLS; this is often the case at small and rural health posts. If facilities do not have the equipment to access IPLS, then they will continue to rely on the paper-based method [18]. When the MOH provided tablets to individual facilities, they reported they were more likely to use the electronic components of IPLS. However, even if facilities had the hardware available, if they did not have consistent access to the internet, the electronic systems were still of little use because some features of the technology are unable to work offline*.* These findings, while novel, as they relate to technology specifically tailored for OESCs, also affirm what is found in the current literature surrounding digital health research in low- and middle-income countries, which found that increased access to technology and consistent reliable internet led to increased use of the digital health technology [17,18]. Creating dashboards and electronic components of the IPLS that work offline is crucial because internet access is not always consistent in all parts of Amhara [19]*.* To encourage users to transition from paper-based forms and ensure facilities can leverage the benefits of the electronic system, the central EPSS and MOH should ensure the hubs and individual facilities have the physical supplies necessary to access the government’s technology infrastructure.

Users report the traditional siloed process for ordering medical supplies for each health program or clinical condition was tedious and time-consuming. Having all the supplies needed to manage obstetric emergencies in one place, along with supplies for other medical situations, makes the ordering process more streamlined. Furthermore, having all this information available in one place is useful when pharmacists and supply managers are trying to view inventory levels, determine their consumption, and forecast future needs. Pharmacists stated they enjoy having this information available to them electronically. As a developmental next step, the Ethiopian MOH could take steps to automate the electronic components of IPLS (internet-based and hardware devices) at all health care facilities, which will help with the calculation of monthly consumption, waste, and expiration so that pharmacists can have these current, real-time data available when they are making supply requests to the regional hubs. This recommendation is based on participant reports of calculation errors and is supported by the preexisting literature which found forecasting errors to be a concern with Ethiopia’s medical supply chain [20]. In addition, knowing what is available at various hubs can assist in making supply distribution decisions and advancing supply accessibility across hubs and the facilities they service, which was reported in a study conducted in South Africa [21]. Without the availability of rich inventory data, facilities will continue to remain in the dark about their true readiness to manage basic obstetric emergencies, and the central EPSS and regional hubs will not have the necessary information to provide essential emergency supplies prospectively, before stockouts and maternal deaths occur [20,22].

Facilities reported using the same process for ordering and obtaining supplies was incredibly helpful. Using one system to order all basic emergency obstetric supplies created a uniform communication flow between individual facilities, regional hubs, and central EPSS. However, as the IPLS ages, it will benefit from ongoing updates and adaptation to the evolving needs of the system’s users at multiple levels, which aligns with best practices stating that technology should be routinely modified and updated to both align with user feedback and comply with recent regulatory standards [23]. For example, providing space to justify seasonal differences in supply request quantities would be a direct response to user requests, allowing facilities to explain the change in demand, helping hubs and central EPSS to both forecast future needs based on seasonal trends, and also helping central planners make informed, strategic decisions even in cases where they must distribute a smaller number of supplies to multiple hubs [24]. Furthermore, similar work conducted in Zambia identified seasonal changes to be a driver of malaria medication stockouts in the country; therefore, Ethiopia’s modification to include justification for seasonal differences would also align with the preexisting literature [25].

The greatest concern related to the workflow and communication of the OESC was an inability to accurately forecast future supply needs. This is a critical issue because inaccurate forecasting can lead to undersupplying and stockouts or oversupplying and waste of medication due to expiration. This same issue was identified in a similar study of hospital laboratories in Gambela, a region in the southwest of Ethiopia, which emphasizes that this may be a countrywide issue and not only seen Amhara [26]. When individuals know their true consumption, they can analyze the trend in their past use patterns and forecast their future needs. Increasing data visibility is not only a concern at facilities for preventing maternal deaths but also crucial at every stage of the supply chain to ensure consistent, predictable emergency supply availability for reducing facility-based mortality at the population level in Amhara. Previous research in the United States demonstrated the utility of using multilevel dashboards (ie, facility and national levels) to monitor the distribution and inventory status of COVID-19 vaccines [27]. Currently, the greatest communication breakdown occurs between regional hubs and individual facilities. Bridging the gap between these 2 areas will not only assist with forecasting but also provide an additional level of surveillance at the regional hubs which can monitor inventory levels at facilities and prospectively send out supplies if they notice a facility is reaching critically low levels to prevent stockouts in advance.

Technology updates and changes must be reviewed and monitored not only to measure their success but also to investigate if there are any positive or negative unintended consequences of the technology updates [23]. This is seen in the *Dagu* 1 to *Dagu* 2 updates which improved data transparency within the system but also introduced barriers to efficient use of the system at the facility level. On the basis of user complaints from this update, it is imperative to conduct a formative assessment with end users to ensure the technology updates cohere with the HIT’s intended purpose and existing user workflow [28]. Furthermore, the *Dagu 1* to *Dagu 2* updates underscore the notion that usefulness does not always equal usability, and updates to technology should be reviewed by targeted end users to ensure they will not have unintended negative consequences. This same problem occurred when electronic health records were widely integrated into hospitals in the United States in the 2010s [29]. Before implementing changes within the electronic components of IPLS, those individuals who are leading these updates should establish a process to monitor and measure the impact of the changes.

The findings from these interviews highlight the need to bridge the data visibility gap between facilities and regional hubs. Furthermore, facility-level participants stated that they would appreciate the ability to view inventory data electronically and having that information available would assist in forecasting decisions and supply requests. The discussions with individuals at all levels of the supply chain underscore the importance of having data views that are tailored to the different job types and are supported by the current literature. Previous health technology research has found curated technology displays for different end users or job tasks to be beneficial [30]. Providing health technology, such as dashboards with different views for the various stakeholders in the supply chain is helpful because it can provide a granular view for each stakeholder depending on their information needs and job functions [31]. Different types of dashboards that could be useful would include dashboards for individuals who work at facilities and regional hubs. The individual facility employees can use the dashboards to assist with performing accurate forecasting and supply requests, as well as monitoring their day-to-day inventory levels. In contrast, the dashboards for region hub employees could assist with the hub’s ability to monitor the inventory levels of the facilities under their jurisdiction and provide supplies in advance of stockouts.

One facilitator of the current human-computer interfaces is that there is already strong buy-in from the users because they believe the technology is both useful for their jobs and easy to use. While some reported ease of use issues, most respondents indicated the existing EPSS system and IPLS were usable. New dashboards must maintain these existing usability strengths. Future steps can be taken to curate additional individualized dashboards that provide the information that is most pertinent for each stakeholder across the health system from facilities to regional hubs and the central EPSS. In addition, if individuals continue to use the technology on tablets or mobile devices, then the EPSS could ensure the technology is tailored for use on those devices instead of only computers. This incremental adaptation of the IPLS and EPSS interfaces to mobile technology can ensure that the supply monitoring technology follows usability heuristics for mobile health, which is critical to ensure success of the technology on the new platform [32].

### Limitations

One potential limitation of the study is that when transcripts were translated from Amharic to English, there may have been unintended translation errors. However, to mitigate this concern, a medical anthropologist, who lives in Amhara and has >14 years of experience performing qualitative data collection and translating data from Amharic to English, performed the translations. In addition, while this work was designed for health care facilities in the Amhara region of Ethiopia, all regional- and facility-level participants worked in Bahir Dar city, the capital of the region. This limitation on sampling outside the regional capital was due to civil unrest which prevented team members from safely visiting other locations [33]. Despite this potential limitation, the study sample represents different health care facility levels and regional positions and still provides a rich understanding of the current OESC dynamics in Amhara. Finally, the sample was predominately male, which may have unintentionally excluded the opinions and experiences of female individuals working within the OESC. However, this male-dominated sample matches preexisting HIT user staffing in the region.

### Conclusions

This study captured insight into the information and communication flow within Amhara’s OESC. Participant responses identified several strengths and barriers related to the success of the current IPLS. A frequently noted strength was the high degree of integration of medications and supplies that can be purchased through the system. Common barriers include a lack of data transparency at the facilities and also between facilities and regional hubs. The findings offer several recommendations for how future technology can be designed and tailored to meet the needs of current OESC employees. In addition, the results of the interviews underscored the importance of conducting qualitative research early in the developmental process of HIT to ensure a rich understanding of the current environment and user tasks that the technology will need to accomplish. Although these data come from Amhara and national MOH informants, the EPSS system and IPLS are common across Ethiopia’s regions. Consequently, HIT-based innovations resulting from this research can be readily adapted to the hubs and facilities in multiple Ethiopian regions.

## Appendix

Multimedia Appendix 1 Qualitative interviews guide for semistructured interviews with obstetric emergency supply chain employees in Amhara, Ethiopia: English version.

Multimedia Appendix 2 Qualitative codebook for semistructured interviews with obstetric emergency supply chain employees in Amhara, Ethiopia.

Multimedia Appendix 3 Thematic saturation table for qualitative semistructured interviews with obstetric emergency supply chain employees in Amhara, Ethiopia; tracking codes obtained during each data collection session.

Multimedia Appendix 4 Study participant demographics from semistructured qualitative interviews with obstetric emergency supply chain employees in Amhara, Ethiopia.

Multimedia Appendix 5 Additional Sociotechnical Model dimensions not included in the main manuscript from the qualitative interviews with facility-, regional-, and federal-level obstetric emergency supply chain employees during qualitative semistructured interviews in Amhara, Ethiopia.

Multimedia Appendix 6 Data sources and communication channels that were identified as currently available to facility-, regional-, and federal-level obstetric emergency supply chain employees during qualitative semistructured interviews in Amhara, Ethiopia.

Multimedia Appendix 7 Human-computer interfaces that were reported as currently available to facility-level, regional-level, and federal-level obstetric emergency supply chain employees during qualitative semistructured interviews in Amhara, Ethiopia.

Multimedia Appendix 8 Reported barriers and facilitators for the human-computer interface dimensions (all aspects of the digital application that the user can see, touch, hear, or manipulate) of the Sociotechnical Model for Studying Health Information Technology in Complex Adaptive Healthcare Systems extracted from semistructured interviews with obstetric emergency supply chain employees in Amhara, Ethiopia.

## Abbreviations

BEmOC: basic emergency obstetric care needs
EPSS: Ethiopian Pharmaceutical Supply Service
HIT: health information technology
IPLS: integrated pharmaceutical logistics system
MMR: maternal mortality ratio
MOH: Ministry of Health
OESC: obstetric emergency supply chain
RRF: requesting and reporting form

## Data Availability

The data sets generated during and analyzed during this study are available from the corresponding author on reasonable request.
