# Supplementary material for: Obstetric Emergency Supply Chain Dynamics and Information Flow Among Obstetric Emergency Supply Chain Employees: Key Informant Interview Study
Source: JMIR Form Res. 2024 Sep 5;8:e59690. doi: 10.2196/59690 (PMC11413542; doi:10.2196/59690)
Supplement: Multimedia Appendix 1 [file formative_v8i1e59690_app1.docx]

# Multimedia Appendix 1. Qualitative interviews guide for semi-structured interviews with obstetric emergency supply chain employees in Amhara, Ethiopia: English version

**PROTOCOL TITLE**: Creation of an Electronic Dashboard to Enhance the Obstetrics Emergency Supply Chain in Amhara, Ethiopia

The purpose of this interview is to explore the causes of obstetric emergency supply stockouts, Amhara’s current health system approaches to restocking these supplies, and information flow dynamics that impact ordering and shipping of obstetric emergency medical supplies. We are interested in hearing your opinions and learning about your personal experiences. There are no right or wrong answers, we are interested in your honest opinion, and everything you say during this interview will be kept confidential.

1. What is your DOB?
2. What is your job title?
3. Years of experience working with Ethiopia’s EPPS?
4. Can you describe your process for ordering (or shipping) obstetric emergency supplies? (Barriers and facilitators in the process, how often it occurs, who does this task, was there any training, decision making process)
5. What do you see as the major challenges to having the right supplies on hand to deal with obstetric emergencies when they occur? (Communication breakdowns, frequently unable to obtain certain items, computers available and/or consistent wifi)
6. What are your impressions of the paper-based supply request system? (Likes, dislikes, barriers and facilitators for use, areas of improvement)
7. Can you describe your experience using the IPLS system? (Likes, dislikes, barriers and facilitators for use, areas of improvement, training if it occurred)
8. What type of data would make healthcare facilities more effective at requesting the appropriate around of medical supplies?
9. What are the most important things to consider when determining where and how much supply you (federal/regional) ship out?
10. Do you find the current dashboards useful for your task completion? Please explain
11. Is there anything else that you’d like me to know about the topics that we’ve discussed today that I didn’t ask about?
