# Supplementary material for: Obstetric Emergency Supply Chain Dynamics and Information Flow Among Obstetric Emergency Supply Chain Employees: Key Informant Interview Study
Source: JMIR Form Res. 2024 Sep 5;8:e59690. doi: 10.2196/59690 (PMC11413542; doi:10.2196/59690)
Supplement: Multimedia Appendix 2 [file formative_v8i1e59690_app2.docx]

| **Code/Domain** | **Subcodes** | **Definition** | **When to apply** | **When not to apply** |
| --- | --- | --- | --- | --- |
| Hardware and Software | General | The digital infrastructure and equipment used to operationalize the clinical application explored | - When participants describe the physical equipment and digital programs needed to accomplish tasks related to monitoring facility readiness to manage obstetric emergencies | - When participants describe components outside of the digital infrastructure or physical equipment needed to monitor facility readiness to manage obstetric emergencies |
|  | Facilitator |  | - When participants describe physical equipment and digital programs that make it easier for them to monitor facility readiness to manage obstetric emergencies | - When participants describe components outside of the digital infrastructure or physical equipment needed to monitor facility readiness to manage obstetric emergencies - When participants describe the digital infrastructure or physical equipment and how make it harder to accomplish their tasks |
|  | Barrier |  | - When participants describe physical equipment and digital programs that make it harder for them to monitor facility readiness to manage obstetric emergencies | - When participants describe components outside of the digital infrastructure or physical equipment needed to monitor facility readiness to manage obstetric emergencies - When participants describe the digital infrastructure or physical equipment and how they make it easier to accomplish their tasks |

**Multimedia Appendix 2.** Qualitative codebook for semi-structured interviews with obstetric emergency supply chain employees in Amhara, Ethiopia

| Clinical Content | General | The categorical or numerical data and images that make up the “language” of the clinical application | - When participants describe the data, images, and labels needed to develop the language of the clinical application | - When participants describe things beyond the data, images, and labels needed to develop the language of the clinical application |
| --- | --- | --- | --- | --- |
|  | Facilitator |  | - When participants describe data, images, and labels that make it easier to develop and/or understand the language of the clinical application | - When participants describe things beyond the data, images, and labels needed to develop the language of the clinical application - When participants describe the data, images, and labels that make it harder to develop and/or understand the clinical application |
|  | Barrier |  | - When participants describe data, images, and labels that make it harder to develop and/or understand the language of the clinical application | - When participants describe things beyond the data, images, and labels needed to develop the language of the clinical application - When participants describe the data, images, and labels that make it easier to develop and/or understand the language of the clinical application |
| Human-Computer Interface | General | All aspects of the digital application that the user can see, touch, hear, or manipulate | - When participants are describing the things that they can see, touch, hear, or manipulate within the digital application | - When participants describe components outside of those that they can see, touch, hear or manipulate |
|  | Facilitator |  | - When participants describe how the components that they can see, touch, hear, or manipulate make it easier to accomplish their tasks | - When participants describe components outside of those that they can see, touch, hear or manipulate - When participants describe how the components that they can see, touch, hear, or manipulate make it harder to accomplish their tasks |
|  | Barrier |  | - When participants describe how the components that they can see, touch, hear, or manipulate make it harder to accomplish their tasks | - When participants describe components outside of those that they can see, touch, hear or manipulate - When participants describe how the components that they can see, touch, hear, or manipulate make it easier to accomplish their tasks |
| People | General | The application users from the developers of the health information technology (HIT) to the end-users | - When participants describe the people who use or are expected to use the HIT | - When participants describe things unrelated to the people who use or are expected to use the HIT |
|  | Facilitator |  | - N/A | - N/A |
|  | Barrier |  | - N/A | - N/A |
| Workflow & Communication | General | The necessary steps that a user must accomplish to complete the task successfully and effectively | - When participants describe the steps they have to accomplish to successfully complete their tasks | - When participants describe things beyond the steps necessary to accomplish tasks successfully |
|  | Facilitator |  | - When participants describe the steps that make it easier to complete their tasks | - When participants describe things beyond the steps necessary to accomplish tasks successfully - When participants describe the steps that make it harder to complete their tasks |
|  | Barrier |  | - When participants describe the steps that make it harder to complete their tasks | - When participants describe things beyond the steps necessary to accomplish tasks successfully - When participants describe the steps that make it easier to complete their tasks |
| Internal Organizational Features | General | The policies, procedures, and culture within the specific organization using the technology | - When participants describe the policies, procedures, rules, and cultures within their own organization that relate to using HIT | - When participants describe things outside of the policies, procedures, rules, and cultures of their own organization that relate to using HIT - When participants describe the policies, procedures, rules, and cultures outside of their organization that relate to using HIT |
|  | Facilitator |  | - When participants describe the policies, procedures, rules, and cultures within their own organization that make it easier for them to use HIT | - When participants describe things outside of the policies, procedures, rules, and cultures of their own organization that relate to using HIT - When participants describe the policies, procedures, rules, and cultures outside of their organization that relate to using HIT - When participants describe the policies, procedures, rules, and cultures within their own organization that make it harder for them to use HIT |
|  | Barrier |  | - When participants describe the policies, procedures, rules, and cultures within their own organization that make it harder for them to use HIT | - When participants describe things outside of the policies, procedures, rules, and cultures of their own organization that relate to using HIT - When participants describe the policies, procedures, rules, and cultures outside of their organization that relate to using HIT - When participants describe the policies, procedures, rules, and cultures within their own organization that make it easier for them to use HIT |
| External Rules and Regulations | General | The policies, procedures, and culture within the larger geographical area where the HIT is located | - When participants describe the policies, procedures, rules, and cultures outside of their organization that relate to using HIT | - When participants describe things not related to the policies, procedures, rules, and cultures outside of their organization that relate to using HIT - When participants describe the policies, procedures, rules, and cultures within their own organization that relate to using HIT |
|  | Facilitator |  | - When participants describe the policies, procedures, rules, and cultures outside of their organization that make it easier to use HIT | - When participants describe the policies, procedures, rules, and cultures within their own organization that relate to using HIT - When participants describe the policies, procedures, rules, and cultures outside of their organization that make it harder to use HIT |
|  | Barrier |  | - When participants describe the policies, procedures, rules, and cultures outside of their organization that make it harder to use HIT | - When participants describe the policies, procedures, rules, and cultures within their own organization that relate to using HIT - When participants describe the policies, procedures, rules, and cultures outside of their organization that make it easier to use HIT |
| Measuring and Monitoring | General | The evaluation process and method of measuring the effectiveness of HIT, including both intended and unintended consequences | - When participants describe the evaluation process for exploring the effectiveness and usefulness of HIT along with the intended and unintended consequences of the technology | - When participants discuss things unrelated to the evaluation process for exploring the effectiveness and usefulness of HIT including intended and unintended consequences |
|  | Facilitator |  | - When participants describe useful evaluation processes for exploring the effectiveness and usefulness of HIT - When participants describe the positive intended and unintended consequences of the technology | - When participants discuss things unrelated to the evaluation process for exploring the effectiveness and usefulness of HIT including intended and unintended consequences - When participants describe difficult or hard to use evaluation processes for exploring the effectiveness and usefulness of HIT - When participants describe the negative consequences of the technology |
|  | Barrier |  | - When participants describe difficult or hard to use evaluation processes for exploring the effectiveness and usefulness of HIT - When participants describe the negative consequences of the technology, including those that were unintended | - When participants discuss things unrelated to the evaluation process for exploring the effectiveness and usefulness of HIT including intended and unintended consequences - When participants describe useful evaluation processes for exploring the effectiveness and usefulness of HIT - When participants describe the positive consequences of the technology |
