# Supplementary material for: Obstetric Emergency Supply Chain Dynamics and Information Flow Among Obstetric Emergency Supply Chain Employees: Key Informant Interview Study
Source: JMIR Form Res. 2024 Sep 5;8:e59690. doi: 10.2196/59690 (PMC11413542; doi:10.2196/59690)
Supplement: Multimedia Appendix 3 [file formative_v8i1e59690_app3.docx]

**Multimedia Appendix 3.** Thematic saturation table for qualitative semi-structured interviews with obstetric emergency supply chain employees in Amhara, Ethiopia; tracking codes obtained during each data collection session

| **Codes and Categories** | **F1** | **F2** | **F3** | **F4** | **F5** | **R1** | **R2** | **R3** | **R4 & R5** | **H1** | **H2** | **H3** | **H4** | **H5** | **H6** | **H7** | **%*** |
| --- | --- | --- | --- | --- | --- | --- | --- | --- | --- | --- | --- | --- | --- | --- | --- | --- | --- |
| Hardware & Software | X | X | X | X |  | X | X | X | X | X | X | X | X | X |  |  | 82% |
| Hardware & Software Barriers | X | X | X | X | X | X | X | X | X | X | X | X | X |  | X | X | 94% |
| Hardware & Software Facilitators | X | X | X | X |  | X | X | X | X | X | X | X |  | X | X | X | 88% |
| Clinical Content | X | X | X | X | X | X | X | X | X | X | X | X | X | X |  | X | 94% |
| Clinical Content Barriers | X |  | X | X |  | X | X | X | X | X | X | X |  | X |  | X | 76% |
| Clinical Content Facilitators | X | X |  |  |  |  |  |  |  | X |  | X | X |  | X |  | 35% |
| Human-Computer Interface | X |  |  | X | X | X |  | X | X | X |  | X |  | X | X | X | 71% |
| Human-Computer Interface Barriers | X |  |  | X |  |  |  | X | X | X |  | X |  |  |  | X | 47% |
| Human-Computer Interface Facilitators | X | X | X |  |  |  | X | X | X | X |  | X |  |  | X |  | 59% |
| People | X | X |  | X | X |  | X | X | X | X | X |  | X | X | X |  | 76% |
| People Barriers | X |  | X |  | X | X | X | X | X | X | X |  | X |  | X | X | 76% |
| People Facilitators | X | X | X | X |  | X | X | X | X |  | X | X | X | X | X | X | 88% |
| Workflow & Communication | X | X | X | X | X | X | X | X | X | X | X | X | X | X | X | X | 100% |
| Workflow & Communication Barriers | X | X | X | X | X | X | X | X | X | X | X | X | X | X | X | X | 100% |
| Workflow & Communication Facilitators |  | X | X | X | X | X |  | X | X | X | X | X | X | X | X | X | 88% |
| Internal Organizational Features | X |  |  | X |  | X | X |  |  | X | X | X |  |  | X |  | 47% |
| Internal Organization Features Barriers |  |  | X | X | X | X |  | X | X | X | X | X | X | X |  | X | 76% |
| Internal Organizational Features Facilitators | X |  |  |  | X |  |  |  | X |  | X |  |  | X |  | X | 41% |
| External Rules & Regulations | X | X |  | X |  | X |  | X |  |  |  | X |  |  |  |  | 35% |
| External Rules & Regulations Barriers | X | X | X | X | X | X | X | X | X | X | X | X | X | X | X | X | 100% |
| External Rules & regulations Facilitators |  | X |  |  |  | X |  |  |  |  | X |  |  |  |  |  | 18% |
| Measuring & Monitoring |  |  |  |  |  | X |  |  | X | X | X |  |  |  |  |  | 29% |
| Measuring & Monitoring Barriers |  |  |  |  |  |  |  |  | X | X |  |  |  |  |  |  | 18% |
| Measuring & Monitoring Facilitators |  |  |  |  |  |  |  |  |  |  |  |  |  |  |  |  | 0% |
| Data Sources | X | X | X | X |  | X | X | X | X | X | X |  |  | X | X |  | 76% |

*Note*. F=federal level respondent, R= regional level respondent, H=healthcare facility level respondent; X= indicates which specific codes were explicated during each data collection sessions; R4 and R5 interviews occurred together during one discussion so the codes that were mentioned during their session are combined

*The percentage of participants mentioning each component
