# Supplementary material for: Obstetric Emergency Supply Chain Dynamics and Information Flow Among Obstetric Emergency Supply Chain Employees: Key Informant Interview Study
Source: JMIR Form Res. 2024 Sep 5;8:e59690. doi: 10.2196/59690 (PMC11413542; doi:10.2196/59690)
Supplement: Multimedia Appendix 4 [file formative_v8i1e59690_app4.docx]

**Multimedia Appendix 4.** Study participant demographics from semi-structured qualitative interviews with obstetric emergency supply chain employees in Amhara, Ethiopia

| **Population Characteristic** | **% (n)** |
| --- | --- |
| Level of the supply chain |  |
| Federal (MOH) | 29.4% (5) |
| Regional (ARHB) | 29.4% (5) |
| Facility | 41.2% (7) |
| Gender |  |
| Male | 82.3% (14) |
| Female | 17.7% (3) |
| Age |  |
| Less than or equal to 35 | 17.6% (3) |
| Between 36-45 | 53.0% (9) |
| 46 and older | 29.4% (5) |
| Years of experience working the supply chain |  |
| 10 years and less | 35.3% (6) |
| Between 11-15 years | 35.3% (6) |
| 16 years or more | 29.4% (5) |
