# Supplementary material for: Obstetric Emergency Supply Chain Dynamics and Information Flow Among Obstetric Emergency Supply Chain Employees: Key Informant Interview Study
Source: JMIR Form Res. 2024 Sep 5;8:e59690. doi: 10.2196/59690 (PMC11413542; doi:10.2196/59690)
Supplement: Multimedia Appendix 5 [file formative_v8i1e59690_app5.docx]

## **Multimedia Appendix 5.** Additional Socio-technical model dimensions not included in the main manuscript from the qualitative interviews with facility-level, regional-level, and federal-level obstetric emergency supply chain employees during qualitative semi-structured interviews in Amhara, Ethiopia

## **People**

It is the responsibility of individual facilities to quantify their consumption and then forecast their upcoming needs. Pharmacists complete the monthly orders with collaboration and assistance from multiple teams at the facility to determine forecasting numbers (i.e., distribution, house inventory management, quantification, marketing). Store managers at individual facilities receive the shipments from their designated hubs and check that they received the requested inventory. It is the role of an individual at the dispensing unit, such as an employee in the maternal child health unit, to internally request supplies from the pharmacist at their facility. Medical directors and health facility managers work together to try and overcome budget constraints so they can order the anticipated supplies from distribution hubs. The following table presents the barriers and facilitators related to people and exemplar quotes from various levels of the system.

**Reported barriers and facilitators for the people dimension of the Socio-technical model for studying health information technology in complex adaptive healthcare systems extracted from semi-structured interviews with obstetric emergency supply chain employees in Amhara, Ethiopia**

| **Component** | **Themes** | **Quotes** |
| --- | --- | --- |
| **People**  *The application users from the developers of the technology to the end-users* | **Barriers** | *“The necessity for ongoing training arises from the fact that when attrition occurs, every health facility employee does not pass on manuals or share their knowledge with the subsequent employee. Attrition is a major issue for health facilities, and the next issue is a capacity deficit.” Federal #1* |
|  | -High attrition rate amongst pharmacists |  |
|  | -Lack of computer literacy at smaller facility level | *“Many of these institutions’ workers lack computer skills so using paper based is advantageous for these situations.” Regional #2* |
|  | -Inconsistent training for application end-users | *“People might not properly complete the quantity request form.” Regional #3*  *“I wish for the manager, or the leader of these activities should not only order activities for other experts to work on but he/she should follow-up how experts are working on all the activities.” Hospital #4*  *“We weren’t given any lessons [training] on how to use the purchasing system. And even in the store, I couldn’t get training on how to store or work with it [supply request process]. Basically, we were all just assigned without any prior knowledge of purchasing or store training.” Hospital #6*  *“Since the practices we were using two or three years ago have changed now and may change in the future, it is important for health care providers to do so based on both national guidelines and international recommendations.” Federal #5* |
|  | -Not all necessary personnel have authorization to use the technology | *“If I want to know what is happening at this institution, I cannot. It means that only authorized people can see it.” Hospital #3* |
|  | **Facilitators** | *“IPLS training is included in the preservice training before the students (Pharmacy) graduated. They will learn about IPLS because training might not be included at the health profession” Federal #3* |
|  | -Pharmacists receive IPLS training before they graduate |  |
|  | -Opportunities for continuing education | *“Virtually all experts have training and experience. Experience is a consideration even during the hiring process. In addition, we provide courses to strengthen the skills of our experts with the assistance of partners after they join us.” Regional #2*  *“Our office [Amhara regional health bureau supply chain office] started improving professional skill of staff members at health facilities through training and follow-up in order to solve such issues.” Regional #3*  *“If there are no trained experts, we send a request to the concerned body to provide the training for them.” Hospital #2* |
|  | -Facility-level expertise | *“Currently, we have recruited several experts to work on supply management” Hospital #4*  *“The medical unit, emergency unit, laboratory unit, and pharmacy unit are some of the departments that make up the MTC [consumption and ordering committee]. The committee’s job is evaluation; it assesses the departments’ requests and prioritizes issues before making final conclusion which happen after thorough discussion among committee members. The cost is taken into account when they make a decision.” Hospital #5* |

## **Internal Organizational Features**

The Ethiopian federal government has mandated that the Ethiopian Pharmaceutical Supply Service (EPSS) play the principal role in delivering and controlling medicine and medical equipment provision throughout the country. Additionally, it is the role of EPSS to negotiate contracts with external suppliers to obtain medical equipment and supplies that cannot be obtained in Ethiopia. It is the job of regional hubs to summarize and consolidate the needs of individual facilities and submit requests to central EPSS for supplies. When problems with orders occur EPSS will reach out to hubs and management at individual healthcare facilities to make them aware of the concern. The following table displays respondent perceptions of barriers and facilitators as well as exemplar quotes.

**Reported barriers and facilitators for the internal organizational features dimension of the Socio-technical model for studying health information technology in complex adaptive healthcare systems extracted from semi-structured interviews with obstetric emergency supply chain employees in Amhara, Ethiopia**

| **Component** | **Themes** | **Quotes** |
| --- | --- | --- |
| **Internal Organizational Features**  *The policies, procedures and culture within the specific organization using the technology* | **Barriers** | *“Maternal services are free, but hospitals do not [receive supplies] for free. There is nobody that replaces what has been purchased after it has been used. So, what will happen to the hospital? The purchasing power is weakening. As it gets weaker, it will not provide proper service at the end…There must be a body to be responsible to cover the costs for maternal healthcare services. But not the hospital, if it is a regional hospital than the regional government should cover [the costs], if it is a district hospital that district office should cover.” Hospital #3* |
|  | -Inability to charge for services |  |
|  | -Supply purchasing restrictions | *“Without the permission of the EPSS, we cannot buy the medical supplies from outside market, even though we have the money to do so.” Hospital #2*  *“The case of the…hospital, we planned for our hospital with an anticipated cost of more than 90 million birr. However, the government has not yet provided this 90 million birr, so the hospital is currently unable to purchase and we are unable to provide important health services that are expected from this hospital.” Hospital #7* |
|  | -The supply chain cannot keep up with the demand of large, busy hospitals | *“We always have problems [preventing stockouts] because our institution is so big and giving services to so many people…We face shortage of medical supplies, equipment, and medicine because we always need and use all these materials every day and we face shortage.” Hospital #4*  *“Although EPSS is a major supplier of healthcare facilities, it is now unable to meet our demands. That is what I noticed and what the staff as a whole reports.” Hospital #5*  *“The internal manufacturers are not able to meet the country’s demand. Assume that is they have the capacity to cover the country’s demand, we can save time, money, and labor. Unfortunately, they cannot afford to satisfy medicine demand.” Regional #5* |
|  | **Facilitators** | *“The Ethiopian government is constantly keen to increase access to healthcare for all inhabitants.” Federal #1* |
|  | -Government-level buy-in to support the implementation and utilization of IPLS |  |
|  | -Strong transportation system | *“The EPSS transports the medical supplies needed [for] obstetric emergency and those needed to give services to the mothers and children, and family planning themselves.” Hospital #2* |
|  | -New reimbursement process | *“The finance system also improved, such as our financing system for example we now use Model 9 for [reimbursement], and where we also use Model 22 to send disbursements.” Regional #1* |
|  | -Central EPSS offsets costs when it can | *“In some areas, the government covers the cost of the mobile data or the internet connectivity.” Federal #2* |

##

## **External Rules and Regulation**

While strides have been made to improve the obstetric emergency supply chain in Ethiopia, progress is still dependent on many influences outside the government health system. Some of these external forces include global markets and pricing, civil unrest, and the availability of funds to purchase medical supplies and medication internationally and within Ethiopia, or variable emergency commodities being provided outside the government system through international organizations such as the World Health Organization or Unicef. The following table summarizes the barriers and facilitators identified for external rules and regulations.

**Reported barriers and facilitators for the external rules and regulations dimension of the Socio-technical model for studying health information technology in complex adaptive healthcare systems extracted from semi-structured interviews with obstetric emergency supply chain employees in Amhara, Ethiopia**

| **Component** | **Themes** | **Quotes** |
| --- | --- | --- |
| **External Rules & Regulations**  *The policies, procedures and culture within the larger geographical location that technology is located* | **Barriers** | *“How can we order and what can we do if there is no budget?” Hospital #1*  *“Some of the medical equipment we require is not always available on the market because it is very expensive and difficult to obtain.” Hospital #2*  *“The major challenges [of] the availability it starts from the national level because we usually quantify the national requirement every year but because of the budget shortage we didn’t procure all the commodities and all the requirements based on our available budget.” Federal #3*  *“When we purchase from a private provider/vendor, there is another problem: cost variance. The price differences between public and private supplies are very obvious. We can significantly reduce costs if we can quickly and easily get what we need from the government vendors. So, in my opinion, the main problem is that supplies from government-owned providers are not available as needed.” Hospital #5*  *“The majority of people in the area use their own data, which they pay for themselves.” Federal #2* |
|  | -Supply cost |  |
|  | -Scarcity of products available on the global market | *“A surgeon’s glove is essential, but there is a lack of them in our hospital because they are not readily available for purchase. As a result of the supply shortfall, we are currently not providing the desired level of service.” Hospital #5*  *“The majority of the time, a delay happens when the necessary supplies are not available on the market.” Hospital #5* |
|  | -Contractors that do not fulfill requests in a timely manner | *“The majority of the supplies for our healthcare facilities come from abroad. For instance, after receiving the contractual agreement, some of the contracted agencies might delay in delivering the goods on time, and others might violate the agreement, which could lead to a lack of items for healthcare facilities.” Regional #3* |
|  | -Geographically difficult to reach locations and transportation difficulties | *“Sometimes, there is a transportation problem, especially for remote health facilities and hospitals that are too far away to take the supplies on time.” Regional #1*  *“Transporting goods is a challenging undertaking; the majority of the time, there are delays in shipping these obstetric emergency supplies.” Hospital #7* |
|  | -Civil unrest disrupting the supply chain | *“The…zone [in Amhara] has security concerns, which adds another hurdle to the shipping of obstetric emergency supplies.” Regional #2* |
|  | **Facilitators** | *“We do not have a transportation problem.” Hospital #2* |
|  | -Strong transportation infrastructure nationally |  |
