# Supplementary material for: Obstetric Emergency Supply Chain Dynamics and Information Flow Among Obstetric Emergency Supply Chain Employees: Key Informant Interview Study
Source: JMIR Form Res. 2024 Sep 5;8:e59690. doi: 10.2196/59690 (PMC11413542; doi:10.2196/59690)
Supplement: Multimedia Appendix 6 [file formative_v8i1e59690_app6.docx]

**Multimedia Appendix 6.** Data sources and communication channels that were identified as currently available to facility-, regional-, and federal-level obstetric emergency supply chain employees during qualitative semistructured interviews in Amhara, Ethiopia.

| **Source** | **Description** | **Format** | **Update Frequency** |
| --- | --- | --- | --- |
| Bin Card | A form kept in the pharmacy with the supplies and used at individual healthcare facilities to track stock on hand, quantity issued/received, losses and adjustments, average monthly consumption, batch number and expiry date  The bin card also reports product name, strength and dosage, unit of issue, and product group | Paper-based | 1 month and when products are issued/received or transferred to another facility |
| Cost Analysis Worksheet | Form sent by healthcare facilities along with RRFs to adjust and prioritize supply requests based on budget availability. The worksheet includes the original request with quantities needed to reach maximum level, unit price, and total price, along with the adjusted request reporting quantities needed to reach maximum level, unit price and total price after prioritizing items to fit the budget. | Electronic or paper-based | 1-2 months |
| Dagu | Digital system used at the facility level that manages both the health program and revolving commodities. The system has dashboards displaying key performance indicators for health services, such as the number of antenatal visits, newborn deliveries, and human resource information. | Electronic | As needed |
| Delivery/ Distribution Invoice and Stock Transfer Voucher (STV) | Forms created by the central level of EPSS that are shipped with the requested supplies to hubs and healthcare facilities reporting what is included in the shipment. | Paper-based | 1 month^1^ |
| Drug list | All medication that should be available at a healthcare facility or regional hub. The list includes drug names, unit of measurement, serial number, expiration date, lot number from its shipment, manufacturer, and price. | Electronic or paper-based | As needed |
| Integrated Pharmaceutical Logistic System (IPLS) | National system for ordering and receiving medical supplies. The system contains most maternal commodities, and electronic components of the system can show consumption, forecasting, and requested supply information. | Electronic or paper-based | Every month |
| Internal Request and Reporting Form (IRRF) | Form completed by each department and laboratory to the main pharmacy reporting stock on hand, quantity received and losses/adjustments, calculated consumption, quantity needed to reach maximum stock status and quantity to be supplied. | Paper-based | Every 2 weeks |
| Report and Request Form (RRF) | Form sent from individual healthcare facilities to regional hubs and from hubs to central EPSS. The forms include the initial balance, stock on hand, delivery report, quantity received, losses and adjustments, ending balance, report of any additional drugs that an institution purchases from another institution, overstock, date of stock out, ordered quantity, products with a shelf-life less than 6 months, and calculated consumption.  This form also reports calculated consumption days out of stock, quantity needed to reach maximum stock status and quantity ordered.  The RRF is also used when a facility receives an order to check if it is the quantity they ordered. | Electronic or paper-based | 1-2 months^2^ |
| Stock Record Card | A form kept in the office of the pharmacy head and used at health centers and hospitals to track stock on hand, quantity issued/received, losses and adjustments, unit price, expiry date and location.  The bin card also reports product name, strength and dosage, unit of issue, and product group | Paper-based | 1 month and when products are issued/received or transferred to another facility |
| Telegram channels | Messaging application used at federal and regional level to convey information | Electronic | As needed |
| Telephone | Healthcare facilities, hubs, and central EPSS will call each other when orders are incorrect/items are missing | N/A | As needed |
| Vitas | Digital system used by the central office of the Ethiopian Pharmaceutical Supply System (EPSS). It has dashboards that are used at the federal and hub level displaying information on the availability of drugs and medical supplies. Central EPSS and hub employees can check the availability of medical supplies. | Electronic | As needed^3^ |

^1^Can also be sent with an emergency stockout request

^2^Can also be sent as an emergency when stockouts occur

^3^ May be updated monthly if hubs and EPSS receive RRFs as a paper-copy or immediately if the forms are sent electronically
