# Supplementary material for: Obstetric Emergency Supply Chain Dynamics and Information Flow Among Obstetric Emergency Supply Chain Employees: Key Informant Interview Study
Source: JMIR Form Res. 2024 Sep 5;8:e59690. doi: 10.2196/59690 (PMC11413542; doi:10.2196/59690)
Supplement: Multimedia Appendix 7 [file formative_v8i1e59690_app7.docx]

**Multimedia Appendix 7.** Human-computer interfaces that were reported as currently available to facility-level, regional-level, and federal-level obstetric emergency supply chain employees during qualitative semistructured interviews in Amhara, Ethiopia.

| Interface description | Central EPSS^a^ office | Regional hub | Facility |
| --- | --- | --- | --- |
| - Bin Cards: Paper - Reports stock on hand, quantities issued and received, losses and adjustments, AMC^b^, batch number and expiry date, product names, strengths and dosage, and product groups | - N/A^c^ | - N/A | - Reports changes in supply quantities - Views inventory data |
| - Drug List: Electronic - Provides information related to drug names, unit of measurement, stock on hand, serial number, expiration date, lot number from its shipment, manufacturer, and price that should be available - Prints reports on the drug information | - Views information on the drugs that should be available | - Views information on the drugs that should be available at the hub level | - Views information on the drugs that should be available at the health care facility^d^ |
| - Drug Lists: Paper - Provides information related to drug names, unit of measurement, stock on hand, serial number, expiration date, lot number from its shipment, manufacturer, and price that should be available | - N/A | - N/A | - Views information on the drugs that should be available at the health care facility |
| - RRF: electronic - Reports stock initial balance, stock on hand, delivery reports, quantity received, losses and adjustments, reports of any additional drugs that an institution purchases from another institution, ending balance, overstock, date of stockout, ordered quantity, and products with a shelf-life <6 months - Supports entry of last month’s consumption - Supports entry of calculated consumption days out of stock - Reports quantity needed to reach maximum stock status - Calculates AMC - Prints paper copies of RRFs | - Views supply requests from regional hubs - Views the number of supplies consumed throughout the country - Views forecasted supply needs for the next several months - Tracks how much stock was shipped to each hub - Views AMC | - Views supply requests from individual health care facilities - Reports consumption rates - Reports calculated consumption days out of stock, and quantity needed to reach maximum stock status - Reports quantity ordered for the upcoming period - Tracks how much stock has been shipped to individual facilities - Views AMC | - Views supply availability^d^ - Views expiration status of supplies - Reports consumption rates - Reports calculated consumption days out of stock, and quantity needed to reach maximum stock status - Reports quantity ordered for the upcoming period - Views AMC |
| - RRF: Paper - Reports stock initial balance, stock on hand, delivery reports, quantity received, losses and adjustments, reports of any additional drugs that an institution purchases from another institution, ending balance, overstock, date of stockout, ordered quantity, and products with a self-life <6 months - Supports entry of last month’s consumption - Supports entry of calculated consumption days out of stock - Reports quantity needed to reach maximum stock status - Reports quantity ordered - Reports AMC | N/A | - Views supply requests from individual health care facilities - Views AMC | - Views supply availability - Views expiration status of supplies - Reports consumption rates - Reports calculated consumption days out of stock, and quantity needed to reach maximum stock status - Reports quantity ordered for the upcoming period - Views AMC |
| - Stock Status Dashboard: Electronic - Calculates expiration status and stock quantity levels - Prints reports related to supply availability and expiration status - Offers a color-coded view of stock availability (eg, overstock, normal, below minimum, emergency order point, and stockout) | - Views inventory data at the central level - Views inventory data at the hub level - Views how many supplies have been ordered | - Views inventory data at the user’s hub - Views inventory data at other hubs in the region - Views how many supplies have been ordered | - N/A |
| - Stock Record Card: Paper - Reports stock on hand, quantity issued/received, losses and adjustments, unit price, expiry date, product name, strength and dosage, and product group | - N/A | - N/A | - Reports changes in supply quantities - Views inventory data |

^a^EPSS: Ethiopian Pharmaceutical Supply Service.

^b^AMC: average monthly consumption.

^c^N/A: not applicable.

^d^Not available or used at all facilities.

^e^RRF: requesting and reporting form.
