# Supplementary material for: Obstetric Emergency Supply Chain Dynamics and Information Flow Among Obstetric Emergency Supply Chain Employees: Key Informant Interview Study
Source: JMIR Form Res. 2024 Sep 5;8:e59690. doi: 10.2196/59690 (PMC11413542; doi:10.2196/59690)
Supplement: Multimedia Appendix 8 [file formative_v8i1e59690_app8.docx]

**Multimedia Appendix 8.** Reported barriers and facilitators for the human-computer interface dimension (all aspects of the digital application that the user can see, touch, hear, or manipulate) of the Sociotechnical Model for Studying Health Information Technology in Complex Adaptive Healthcare Systems extracted from semi structured interviews with obstetric emergency supply chain employees in Amhara, Ethiopia.

| Themes | | Quotes |
| --- | --- | --- |
| **Barriers** | | |
|  | Gap in data visibility of supply inventory (especially between hubs and health care facilities) | - “Departments/units are unable to determine which supplies are in stock and which are not.” [Facility #1] - “Although they [current dashboards] are helpful, they also have problems with data visibility.” [Regional #3] - “There is no dashboard between health facilities and us. This means it is very difficult to access the health facility data because their data is not visible to us.” [Regional #4] - “One of the main supply chain challenges in our country is end to end data visibility.” [Federal #4] - “It is crucial to create and make available such a dashboard, especially for policymakers, decision-makes, and even health management and for health facilities at all levels. If it is available, everyone can view it and it encourages prompt action.” [Federal #5] |
|  | Perceived low ease of use | - “Most of them [facility-level employees], you know, are not doing good with DAGU2 [facility-level software] due to the complexity of the system and its needs like connectivity.” [Federal #4] - “We have a major problem with the way we use this dashboard, as well as the experience we have with it even though the dashboard is very helpful and useful.” [Regional #3] |
| **Facilitators** | | |
|  | Central EPSS^a^ and regional hubs, and some individual health care facilities can use electronic dashboards to view current inventory levels | - [The current electronic system] can provide the item’s serial number, name, unit of measurement, and expiration date in addition to the quantity available and the average monthly consumption for this month.” [Federal #1] - “We can browse the library [dashboard] and learn what is available in various hubs thanks to this tool [dashboard], and we can utilize the dashboard by making requests by checking the progress of other stocks. It also enables us to produce other reports, including those on expired and those that are waste. Therefore, it is without a doubt, really beneficial.” [Regional #2] - “There is a dashboard between the central and branch offices, we can view each other’s data. To your surprise, we can also see the data of other branches, like Negele Borona [Oromia region] and Arba Minch [Southern Nations, Nationalities and Peoples Region]. The other branches can also see our data.” [Regional #4] - “This system [central and hub dashboards] is very important because we can show and see the products for all branches. For example, if there is a misdistribution of the products and the central EPSS sends all products to only three branches out of the 17 active branches by mistake, this dashboard shows where all the products go, and immediately the central office informs the three institutions to distribute the products to all 17 branches. Similarly, branches can also request products because they know where their products go thanks to this dashboard.” [Regional #4] |
|  | Perceived strong usefulness of the current technology | - “IPLS is a crucial system that helps developing nations like Ethiopia, it helps improve their supply chain management, reduce waste, and increase availability.” [Regional #3] - “Certainly, it [the dashboard] is quite helpful [for task completion]. For instance, it may be used to transfer information, make decisions, check for medicine availability, and it also issues and gives us alarm by flashing a red light when a drug is about to expire.” [Facility #1] - “Without a doubt, it [the current dashboards] is very useful. It saves time, lowers labor costs, helps to determine when items pass their expiration dates, and lowers the rate of waste. In general, it is crucial for controlling.” [Facility #5] - The [electronic] IPLS system is capable of calculating and quantification of supplies more accurately and its capacity to minimize errors in the calculations.” [Facility #4] - “The [IPLS] system is very important because it makes it easier for us to complete our tasks and helps us to understand our responsibilities. If the data is stored clearly and is not blocked, we can easily access it and obtain the items we need, including those we receive, those we want to issue, the people to whom we issue them, and the data we issue. Knowing what is stocked out and what is to expire is helpful. It decreases waste as well.” [Facility #5] |
|  | Perceived high ease of use of the current technology | - “Other than a few amenities, IPLS is a user-friendly system.” [Federal #1] - “In the past, we have faced different challenges in using this electronic system, but currently it is very easy.” [Federal #2] - “Because the computer quantifies things on its own, if it is computerized, it makes things very simple.” [Facility #1] - “There are lots of benefits to using computer system [electronic RRFs], it’s usually way easier to fill out a form using a computer than to do it by hand. Plus, since it’s all done on a computer, the calculations are much more accurate.” [Facility #2] |

^a^EPSS: Ethiopian Pharmaceutical Supply Service.
